# Strengths and Weaknesses of Cancer Pain Management in Italy: Findings from a Nationwide SIAARTI Survey

**DOI:** 10.3390/healthcare10030441

**Published:** 2022-02-25

**Authors:** Marco Cascella, Alessandro Vittori, Emiliano Petrucci, Franco Marinangeli, Antonino Giarratano, Cristina Cacciagrano, Emiliano Simone Tizi, Luca Miceli, Silvia Natoli, Arturo Cuomo

**Affiliations:** 1 Department of Anesthesia and Critical Care, Istituto Nazionale Tumori—IRCCS, Fondazione Pascale, 80100 Naples, Italy; a.cuomo@istitutotumori.na.it; 2 Department of Anesthesia and Critical Care, ARCO ROMA, Ospedale Pediatrico Bambino Gesù, IRCCS, 00165 Rome, Italy; alexvittori@libero.it; 3 Department of Anesthesia and Intensive Care Unit, San Salvatore Academic Hospital of L’Aquila, 67100 L’Aquila, Italy; petrucciemiliano@gmail.com; 4 Department of Anesthesiology, Intensive Care and Pain Treatment, University of L’Aquila, 67100 L’Aquila, Italy; francomarinangeli@gmail.com; 5 Department of Surgical, Oncological and Oral Science (Di.Chir.On.S.), University of Palermo, 90133 Palermo, Italy; antonino.giarratano@unipa.it; 6 Department of Anesthesia Intensive Care and Emergency, Policlinico Paolo Giaccone, 90127 Palermo, Italy; 7 Italian Society of Anesthesia, Analgesia, Resuscitation and Intensive Care (SIAARTI), 80100 Rome, Italy; cristina.cacciagrano@siaarti.it (C.C.); emiliano.tizi@siaarti.it (E.S.T.); 8 Unit of Pain Medicine, IRCCS Centro di Riferimento Oncologico (CRO), 33081 Aviano, Italy; luca.miceli@cro.it; 9 Department of Clinical Science and Translational Medicine, University of Rome Tor Vergata, 00173 Rome, Italy; silvia.natoli@uniroma2.it

**Keywords:** cancer pain, survey, breakthrough cancer pain, pain management, chronic pain, opioid, neuropathic pain

## Abstract

Objectives: Despite guidelines, a large percentage of cancer patients continue to suffer from ineffectively treated pain. The authors undertook this survey to assess the strengths and weaknesses of cancer pain management in Italy. Design: This was a prospectively administered survey. Participants: The participants were anesthesiologists of the Italian Society of Anesthesia, Analgesia, Resuscitation and Intensive Care (SIAARTI). Intervention: A 58-item questionnaire covered the demographics and features of cancer pain management in the Italian context. Results: The authors received responses from 611 pain therapists of 279 centers. Only 22% of physicians are exclusively pain therapists. Seventy-five percent are specialists in anesthesiology, intensive care, and pain medicine. Most pain centers are hospital or university facilities (78%). The strengths of cancer pain management in Italy are the careful opioid prescriptions, the use of strategies for the treatment of neuropathic pain, patient/healthcare provider partnerships, and breakthrough cancer pain management. Weaknesses to be addressed include poor adherence to guidelines, inadequate attention toward the patient’s quality of life, insufficient use of minimally invasive techniques, lack of teamwork approaches, inappropriate timing of pain specialist engagement, and poor telemedicine use. Conclusions: Despite several strengths, further efforts are needed to improve the care of patients suffering from cancer pain in Italy.

## 1. Introduction

Despite improvements in the understanding and treatment of cancer pain, and the many international and local guidelines developed on the topic [1,2,3,4], in a substantial percentage of patients, pain is not appropriately treated. Notably, an evidence-based medicine analysis calculated that approximately one-third of cancer patients had inadequately controlled pain, with low-quality control reaching nearly 70% of cases [5]. Since the stage of the disease is a significant variable, a meta-analysis found pain problems in about 66% of patients with advanced and metastatic cancer [6]. Unfortunately, the authors did not observe substantial progress since their previous report in 2007 [7]. This severe gap reflects important clinical implications for patients and in healthcare [8].

There are numerous barriers to adequate pain management. According to previous studies, these barriers include a lack of knowledge, inadequate assessment and treatment, [9] as well as organizational factors, together with system and regulatory obstacles [10].

Overcoming the barriers to proper pain treatment requires immediate attention. Individual researchers and scientific societies commonly debate issues, such as quality of life, treatment compliance, and other unmet needs. Nevertheless, to implement corrective measures, intensify care networks, and fill knowledge gaps, we must go deeper into the problem. Necessarily, the argument must be contextualized according to local realities.

On these premises, the 2019–2021 study group for cancer pain of the Italian Society of Anesthesiology, Analgesia, Resuscitation and Intensive Care (SIAARTI) conducted a nationwide survey to investigate the strengths and weaknesses of cancer pain management.

## 2. Materials and Methods

### 2.1. Questionnaire

Based on the analysis of the literature and from what was highlighted by recent guidelines on the topic, two subgroups of the SIAARTI study group for cancer pain independently formulated two series of “issues of concern” related to the inadequate pain management in cancer patients. The issues were discussed during two online meetings attended by the whole study group. The committee established the items for the development of the questionnaire (Table 1).

These issues were structured into a questionnaire (supplementary file). It was comprised of 58 questions addressing the following items: demographics and center features (10 items); clinical aspects and management (42 items). The latter addressed several topics including pain assessment, guidelines use, availability of pain care protocols, drug regimen strategies (e.g., about breakthrough cancer pain, BTcP), management (and prevention) of side effects, multiprofessional pathways, patient-centered approaches, and minimally invasive techniques.

### 2.2. Participants

The survey was carried out across Italy between June and September 2021. Members of the SIAARTI were asked to participate by an email invitation. The online questionnaire was administered through a computer-aided web interview (CAWI) using the free software, SurveyMonkey (Momentive, San Mateo, CA, USA). No exclusion/inclusion criteria were used as the scope of the study was to obtain an overview of the national scenario. Answers were collected on an anonymous basis.

The survey was conducted in compliance with the European Pharmaceutical Market Research Association (EphMRA) code of conduct. All participants in the survey provided voluntary, informed consent to data collection and use, based upon a clear understanding of the purpose of the data collection.

### 2.3. Statistical Analysis

Results are shown with standard descriptive statistics. Parameters include mean and standard deviation (SD), median and interquartile range (IQR), or proportion of categorical variables as appropriate. For the comparison of the frequency distributions of several items expressed on Likert scales (never, rarely, sometimes, often, all the time), we constructed an indicator that considers the variability of the distributions without altering the nature of the given answers. The proposed indicator is based on a measure of the distance between an observed distribution and a theoretical distribution under optimal conditions, i.e., maximum concordance for the best judgment. Analyses were performed using the RStudio software (RStudio, Boston, MA, USA).

## 3. Results

### 3.1. Demographics and Center Features

Through this investigation, SIAARTI reached and surveyed 279 centers that deal with cancer patients throughout Italy. Table 2 shows demographics and center features.

**Figure 1:**
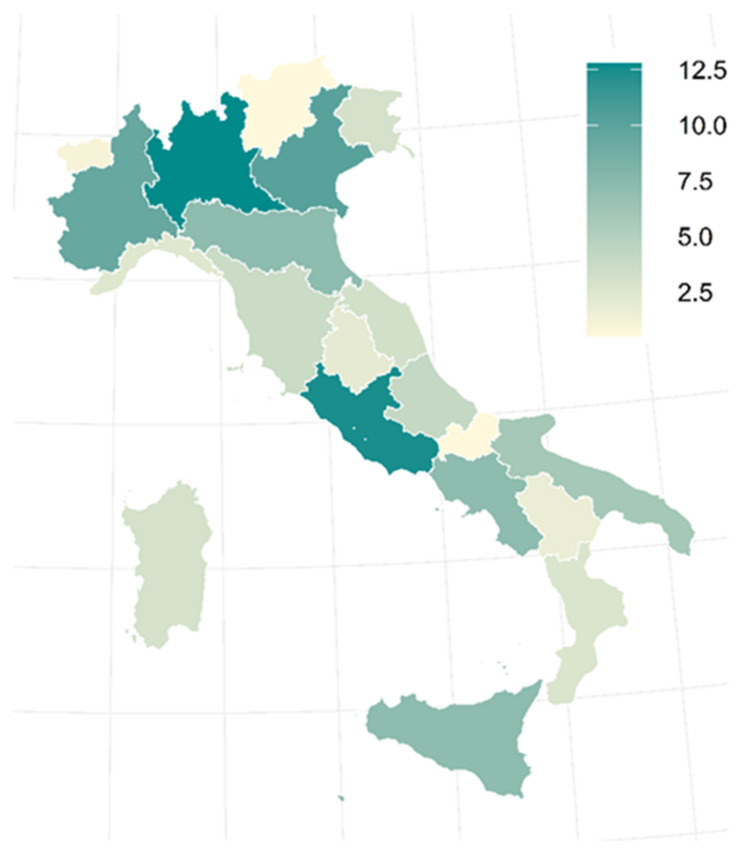
Regional distribution of cancer pain therapists. The color intensity expresses the percentage of respondents of each region.

The regional distribution of physicians involved in cancer pain management is shown in Figure 1. A greater number of pain therapists work in the Lombardy and Lazio regions: 76 (13%) and 74 (12%), respectively.

Following the scope of the study, the results of the items on clinical aspects and management are presented in terms of strengths and weaknesses.

### 3.2. Strengths

**Figure 2:**
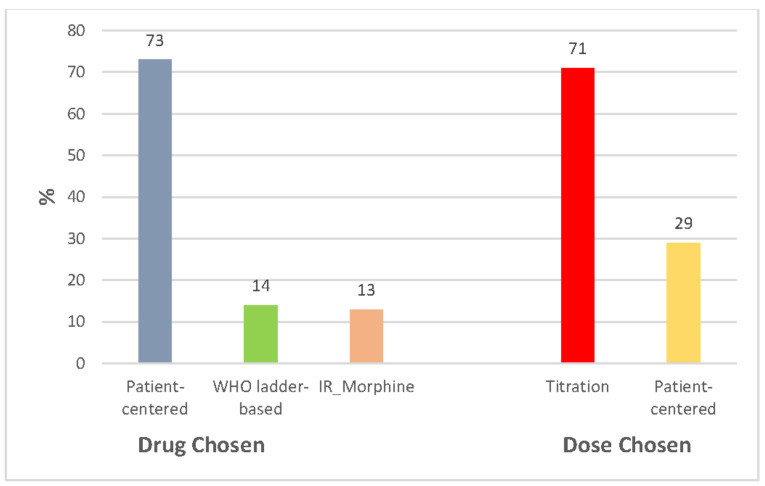
Opioid therapy in naïve patients. Abbreviation: IR, immediate-release.

**Opioid therapy in naïve patients**. In opioid-naïve patients, 73% of specialists declared to choose the type of opioid (weak vs. strong, and agent) based on the patient’s clinical context and pain features; 14% refer to the WHO analgesic ladder; 13% always use immediate-release morphine. Regarding doses and regimens, 29% start with a dose they deem appropriate by considering pain intensity, comorbidities, age, and other variables; 71% perform opioid titration (Figure 2).

**Strategies for treatment of neuropathic pain**. One-third of responders always followed recommendations and/or guidelines (34%), 2% never, 4% rarely, 13% sometimes, and 47% often. Furthermore, more than 50% adopted algorithms (17% always, 40% often).

**Communication strategies and active participation of patients and caregivers in treatment decisions**. Ninety-seven percent of respondents paid special attention to the communication of treatment options and their potential side effects. Regarding the active participation of patients, 91% of clinicians (56% always and 35% often) affirmed that they involved patients in the decision-making process. On the contrary, less than 1% never considered patient engagement, and 8% rarely performed it. Regarding caregivers’ participation, 84% of pain therapists encouraged their involvement (56% always and 35% often).

**Figure 3:**
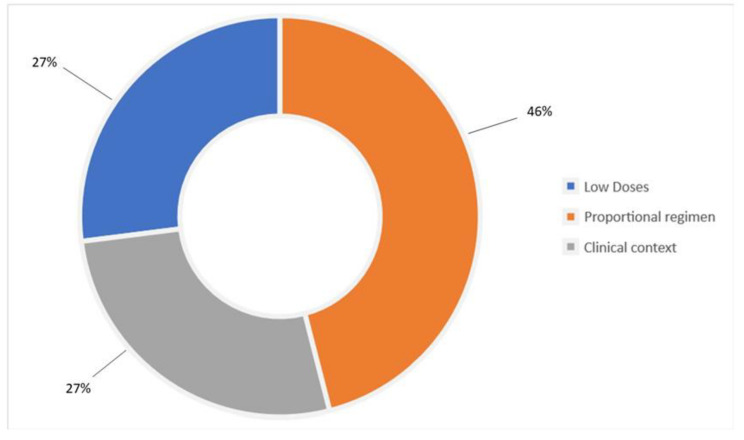
Opioid regimen adopted for breakthrough cancer pain treatment.

**Breakthrough cancer pain (BTcP) management**. Eighty-nine percent of pain therapists manage BTCP through strong opioids. Seventy percent use oral or nasal transmucosal fentanyl formulations (rapid-onset opioids, ROOs). Half of these, administer ROOs when morphine milligram equivalents (MME) are more than 60 mg/day. Fourteen percent use short-acting opioids (e.g., oral morphine). Regarding the opioid regimen adopted for BTcP treatment, 27% start from low dosages; 46% choose the dose of opioids used for background pain (proportional regimen); 27% choose the dose according to the clinical context (Figure 3).

### 3.3. Weaknesses

**Figure 4:**
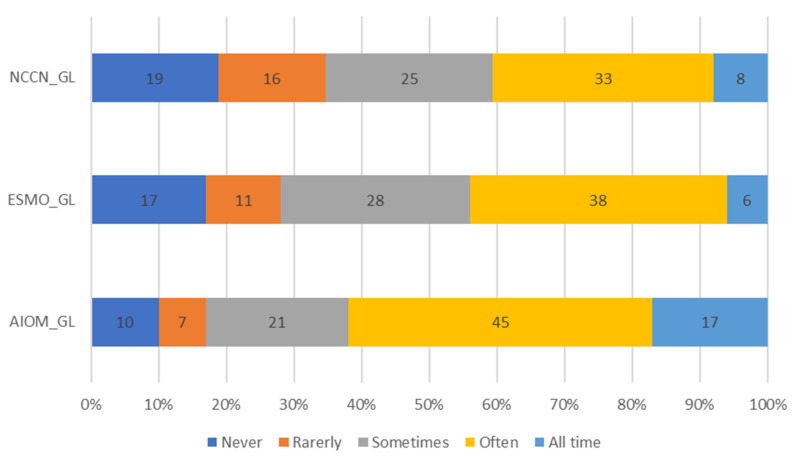
Adherence to guidelines. Legend: NCCN, National Comprehensive Cancer Network; ESMO, European Society for Medical Oncology; AIOM, Italian Association of Oncologists; GL, guidelines.

**Adherence to guidelines.** The adherence to international and Italian guidelines is presented in Figure 4. The American National Comprehensive Cancer Network (NCCN) guideline [2] is always followed by 8% of clinicians; the European Society for Medical Oncology (ESMO) guidelines [1] by 6%; the Italian Association of Oncologists (AIOM) guidelines [4] by 17%.

**Figure 5:**
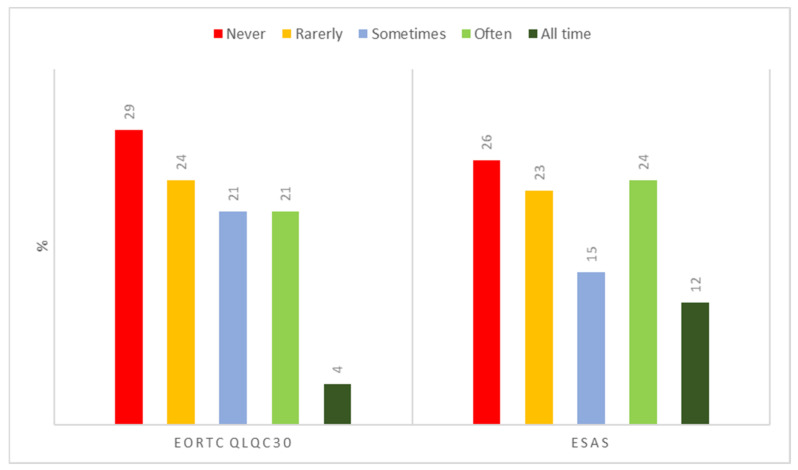
Quality of life questionnaires. Legend: EORTC QLQ-C30, European Organization for Research and Treatment of Cancer QLQ-C30; ESAS, Edmonton Symptom Assessment System.

**Quality of life assessment**. Four percent of clinicians adopt the European Organization for Research and Treatment of Cancer (EORTC) QLQ-C30 questionnaire [11] in each consultation; the Edmonton Symptom Assessment System (ESAS) tool [12] is always used by 12% of the pain therapists (Figure 5).

**Minimally invasive techniques.** Thirty-six percent of the pain therapists very rarely use minimally invasive techniques or invasive procedures.

**Team working.** Fifty-two percent do not follow precise pathways of professional collaboration; multidisciplinary and interdisciplinary strategies are adopted, respectively, by 28% and 20% of the participants.

**Pain therapist involvement.** Fifty-three percent of clinicians serving as pain therapists are involved when pain is difficult to manage; 12% when Numerical Rating Scale (NRS) > 7; 13% in the advanced stages of the disease; 1% when the patient requires high doses of opioids. Twenty-one percent manage pain regardless of the degree of the disease and pain intensity.

**Telemedicine.** Only 18% of clinicians use telemedicine; 20% declared that they are planning to implement this strategy; 62% do not use remote assistance.

## 4. Discussion

This study is a part of an ambitious project of the SIAARTI which aims to bridging the gaps in pain medicine in the Italian context. A previous study by the SIAARTI explored non-cancer pain [13], and selected members of the Society addressed the issue of pain management during the COVID-19 pandemic through a Delphi methodology [14].

Several international and national guidelines are available for helping in cancer pain management. Scientific societies such as the American NCCN [2], the European ESMO [1], and the Italian AIOM [4] update their guidelines regularly. Nevertheless, in our survey, poor adherence to guidelines is a limitation that must be necessarily stressed. Importantly, cancer pain physicians always adopt the AIOM guidelines in only 17%, whereas the European and American guidelines are always followed by less than 10% of respondents (6% and 8%, respectively). On the contrary, specific guidelines and/or recommendations for the treatment of neuropathic pain are particularly followed. Cancer pain is often mixed in nature and expresses an important neuropathic component. Since it represents a great challenge for pain therapists, they are probably more prone to follow strict strategies.

The Italian Scientific Society of Anesthetists must investigate the necessary obstacles (e.g., lack of knowledge) to guideline adherence and interventions to improve it, for example by implementing the training processes. Moreover, the effects of this poor adherence need to be further investigated, especially in terms of patient outcomes.

Opioids remain a cornerstone in cancer pain therapy, but their careful use is mandatory. In opioid-naïve patients, the choice of the drug (weak vs. strong), its dose, and regimen, depend on many factors. In 1986, the WHO developed the famous analgesic ladder and updated it a decade later [15,16]. The developers affirmed that, through the application of the concept “the right drug in the right dose at the right time”, pain relief can be achieved in up to 90% [16]. Unfortunately, these results remain a utopia and the validity of the original ladder has been repeatedly contested [17]. Most likely, a dynamic, multidimensional, and personalized approach for the opioid-naive patient is a strategy that allows calibrating the therapy to the patient’s needs [18,19]. Interestingly, our survey highlighted that, in naive patients, analgesic therapy (i.e., type of opioid) is often managed by starting from an accurate clinical evaluation based on the characteristics of the pain and the clinical setting. On the other hand, opioid titration remains the preferred strategy to establish the more suitable drug regimen.

Objectively measuring the quality of life allows clinicians to manage not only pain, but multiple aspects related to the patient’s functionality. A careful assessment through updated instruments [11,12] can ensure targeted and personalized interventions. Although an exhaustive evaluation of quality of life may require greater commitment and longer visit times, using tools for quality of life assessment is of pivotal importance. Our analysis found that clinicians focus on the symptom “pain”, without evaluating the countless physical, social, and psychological aspects in which it is contextualized. This negative result of the survey must be analyzed by the Scientific Society. Cancer pain, in fact, is the result of the combination of nociception processes with multiple no-nociceptive factors that do not necessarily involve activations of the nociceptive system. Nevertheless, the no-nociceptive elements are closely linked to alterations in quality of life. Therefore, a loop is established between the deterioration of quality of life and pain. In this loop, pain progressively limits the functionality of the individual and, at the same time, the progressive worsening of quality of life amplifies the pain through psychological, social, biological factors.

To ameliorate the quality of care they provide, clinicians should consider their patients’ preferences. Furthermore, active patient participation is of fundamental importance for enhancing the results of pain treatments [20]. The therapeutic alliance presupposes the strengthening of communication strategies and special attention to the patient’s perspective [21]. Although these goals are often difficult to achieve, in our survey, most respondents affirm that they involve patients in the therapeutic choices, developing patient/healthcare provider partnerships. Furthermore, careful communication on pharmacological strategies and potential side effects of drugs is pursued by most clinicians.

Among pain phenomena, BTcP is a sudden exacerbation of pain despite adequate control with opioid therapy [22]. It is a frequent condition affecting up to 70% of cancer patients [23]. Since BTcP is associated with negative outcomes [24], its proper management through rescue doses of strong opioids is needed [25].

Cancer pain management requires the involvement of multidisciplinary or interdisciplinary team working. Clinicians from different disciplines, psychologists, psychiatrists, nurses, physical and occupational therapists, and other professionals can work in parallel (multidisciplinary pathways) or through an integrated approach (interdisciplinary strategies) [26]. These approaches incorporate a holistic biopsychosocial model for pain management [27]. Consequently, since a lack of professional collaboration was found in up to half of the participants, this serious gap requires corrective maneuvers.

Among the multimodal approach to cancer pain, minimally invasive techniques are of key importance. Intrathecal analgesia, peripheral and central neuromodulation, blocks, and other approaches are important resources, especially in complex pain that cannot be managed through exclusively pharmacological approaches. Moreover, the combination of drug therapy with non-pharmacological strategies underpins a personalized, multicomponent approach to pain. Unfortunately, the survey showed that pain therapists must necessarily consider the advantages of invasive and non-invasive procedures, even in the field of cancer pain.

Another gap that we highlight is the inappropriate timing of pain specialist engagement. Remarkably, only one in five pain therapists intervenes, regardless of the degree of the disease and the intensity of the pain. In most cases, the intervention of the specialist is carried out when the treatment becomes extremely complex. In this context, the management of cancer pain and its multiple clinical and functional expressions reduces the possibility of obtaining sufficient results. Scientific societies must bridge the gap by structuring shared pathways and intensifying ad hoc training programs.

Finally, telemedicine is a great chance to simplify continued assistance for patients with chronic pain and enhance their access to care. It is a cost-effective opportunity that can improve treatment adherence [28]. Despite these advantages, only 18% of clinicians involved in cancer pain management use this resource.

## 5. Study Limitations

Our investigation shows several limitations. The data in our possession, even if homogeneous and of quality, only come from the SIAARTI database, which encompasses about 10,000 anesthesiologists. Unfortunately, the precise number of Italian anesthesiologists is not available. Since previous investigations highlighted that in Italy there are about 20,500 anesthesiologists and intensive care unit physicians [29], the target population corresponds to about half of the Italian anesthetists. Although it represents an important selection bias, for the purposes of the survey, this sample appears to be representative. In particular, it is the expression of a scientific society that is authorized by the Ministry of Health to issue guidelines.

Furthermore, other issues such as the management of side effects associated with the use of analgesic drugs needed to be investigated. In the elaboration phase of the survey, the board agreed that an excessive number of items would risk reducing the percentage of participants. Based on these results, the SIAARTI will plan further investigations to gain information and insights into the topic of cancer pain management.

## 6. Conclusions

Although this survey demonstrated important strengths, further efforts are needed to improve the care of patients suffering from cancer pain in Italy. Poor adherence to guidelines, insufficient attention toward the patient’s quality of life, inadequate use of minimally invasive techniques, lack of teamwork approaches, inappropriate timing of pain specialist engagement, and poor telemedicine use are serious issues that need to be urgently addressed.

## Figures and Tables

**Table 1:** Issues of concern.

Inadequate Training Level
Quality of life evaluation
Underuse of opioid pain medications
Underuse of non-pharmacological pain strategies
Barriers that preclude adequate treatment (e.g., fear of opioid addiction)
Poor patient compliance (e.g., communication barriers)
Lack of professional collaboration (multi-professional/interdisciplinary)
Insufficient attention to the diagnosis of specific pain conditions
Patient/healthcare provider partnerships

**Table 2:** Demographics and center features.

Respondents (*n* = 611)	
M/F (*n*)	306/305
Age (Mean ± SD)	51.2 (±10.9)
Expertise	
PS Junior ^	29 (5%)
PS Senior °	18 (3%)
Specialist; <40 years	73 (12%)
41–50 years	171 (28%)
>50 years	322 (53%)
Work activity (%)	
Dedicated pain therapist	22
Anesthesiologist with partial activity as a pain therapist	40
ICU physician with partial activity as a pain therapist	13
Oncologist	3
Radiotherapist	4
Other	17
Health facility (%)	
Hospital/university	78
Home palliative care	3
Research centers	13
Outpatient territorial clinic	2
Hospice	4

Abbreviations: PS = Postgraduate School of Anesthesiology, Intensive Care and Pain Medicine; ICU = intensive care unit. Legend: ^ Junior = I–II year, School of Anesthesiology, Intensive Care and Pain Medicine; ° Senior = III–V year.

## Data Availability

The data that support the findings of this study are available from the corresponding author.

## Supplementary Materials

The following supporting information can be downloaded at https://www.mdpi.com/article/10.3390/healthcare10030441/s1.
