# Supplementary material for: Strengths and Weaknesses of Cancer Pain Management in Italy: Findings from a Nationwide SIAARTI Survey
Source: Healthcare (Basel). 2022 Feb 25;10(3):441. doi: 10.3390/healthcare10030441 (PMC8951760; doi:10.3390/healthcare10030441)
Supplement: Supplementary file 1 [file healthcare-10-00441-s001.zip › healthcare-1582332 - Questionnaire - V1.pdf]

healthcare

Supplementary material

Italian Society of Anesthesia, Analgesia, Resuscitation and Intensive Care (SIAARTI). Cancer Pain Management in Italy - Questionnaire

DEMOGRAPHICS

1.Indicate your mail (optional)

2. Indicate your gender:
□ M
□ F
□ I prefer not to declare it

3. Please indicate your age: _______(years)

4. Indicate the year of Postgraduate Degree in Anesthesiology, Intensive Care, and Pain Medicine:
□ 1970-2021
□ 1st year of Postgraduate School of Anesthesiology, Intensive Care, and Pain Medicine
□ 2nd year of Postgraduate School of Anesthesiology, Intensive Care, and Pain Medicine
□ 3rd year of Postgraduate School of Anesthesiology, Intensive Care, and Pain Medicine
□ 4th year of Postgraduate School of Anesthesiology, Intensive Care, and Pain Medicine
□ 5th year of Postgraduate School of Anesthesiology, Intensive Care, and Pain Medicine

5. Work activity:
☐ Fully pain therapist with postgraduate degree in anesthesiology
☐ Anesthesiologist with partial activity as pain therapist
☐ ICU physician with partial activity as pain therapist
☐ Oncologist
☐ Radiotherapist
☐ Other (specify) ____

6. Specify the role you cover within the unit in which you operate:

□ Trainee
□ Hospital manager
□ Head of Unit (simple unit)
□ Director of UOC (complex unit)
□ Department Director
□ Other (specify) ____

7. Indicate the Italian region in which you mainly carry out your work:
□ Abruzzo
□ Basilicata
□ Calabria
□ Campania
□ Emilia-Romagna
□ Friuli-Venezia Giulia
□ Lazio
□ Liguria
□ Lombardia
□ Marche
□ Molise
□ Piemonte
□ Puglia
□ Sardinia
□ Sicilia
□ Toscana
□ Province of Trento
□ Province of Bolzano
□ Umbria
□ Valle D'Aosta
□ Veneto

8. Indicate the city where you mainly carry out your work

9. Indicate the name of the facility where you mainly carry out your work

10. Indicate the type of health facility where you mainly carry out your work
□ Hospital
□ University Hospital
□ University structure with affiliated assistance activities
□ Private Research Center
□ Public Research Center
□ Classified Hospital (e.g., religious hospitals that are not university)
□ Outpatient territorial clinic (outpatient clinic within the territorial unit not attributable to a hospital)
□ Hospice
□ Home assistance
□ Other (specify)

CLINICAL ASPECTS / CLINICAL MANAGEMENT?

11. How often do you apply the following recommendations/guidelines for cancer pain management?

Italian Association of Oncologists (AIOM) 2019
Management of Cancer Pain in Adult Patients: ESMO Clinical Practice Guidelines- ESMO 2018
NCCN clinical practice guidelines in ONCOLOGY 2019

Never
Rarely
Sometimea
Often
All-time

12. How do you usually manage opioid therapy?
- I refer to analgesic scales (e.g., 0-10 NRS) and I follow a step approach (e.g., WHO ladder)
- In most cases, I use opioids regardless of one-dimensional scales (e.g., NRS)
- I refer to analgesic scales (e.g.,0-10 NRS) but I do not follow a step approach

13. Do you use recommendations/guidelines for the treatment of neuropathic pain?
Never
Rarely
Sometimes
Often
All-time

14. Do you use algorithms for diagnosing neuropathic pain?
Never
Rarely
Sometimes
Often
All-time

15. How do you usually manage pain therapy with minimally invasive techniques?
- I refer to analgesic scales (e.g., 0-10 NRS) and I follow a step approach (e.g., WHO ladder)
- I use a dynamic approach according to the patient's needs and regardless of the analgesic scales (e.g., I plan to treat bone metastases with minimally invasive approaches regardless of the presence of non-severe pain)
- I do not use minimally invasive techniques

16. How do you usually manage analgesic therapy with invasive techniques?
- I refer to analgesic scales (e.g., 0-10 NRS) and I follow a step approach (e.g., WHO ladder)
- I use a dynamic approach according to the patient's needs and regardless of analgesic scales

• I do not use invasive techniques

17. Do you carry out a clinical examination for assessing neuropathic pain?
Never
Rarely
Sometimes
Often
All-time

18. Do you try to involve patients in therapeutic choices?
Never
Rarely
Sometimes
Often
All-time

19. Do you try to involve family members / caregivers in therapeutic choices?
Never
Rarely
Sometimes
Often
All-time

20. Do you explain the problems related to the use of analgesic drugs (side effects)?
Never
Rarely
Sometimes
Often
All-time
21. Do you explain the problems related to the prejudices that often condition patient compliance?
Never
Rarely
Sometimes
Often
All-time

22. Does the assessment of analgesic therapy include the administration of one or more tools aimed at assessing the patient's quality of life?
Never
Rarely
Sometimes
Often

All-time

23. As part of a multi-professional approach, does it include a psychological assessment?
• Yes, with a figure dedicated to pain therapy
• Yes, with non-dedicated figure
• No

24. How do you treat BTCP (spontaneous or induced exacerbation of pain with well-controlled background pain)?
• Preferably through rapid onset opioids/transmucosal fentanyl (ROO) regardless of the type of treatment of the underlying pain
• Preferably ROOs when MME > 60 mg/day
• Preferably NSAIDs
• Preferably short acting opioids (e.g., oral morphine)
• Other ___________

25. When you prescribe an opioid do you also prescribe a treatment for constipation?
• Always, from the first visit
• Secondly, if the patient reports constipation
• Secondly, following the use of validated scales for the assessment of opioid-induced constipation

26. How do you start opioid therapy?
• Titration with immediate-release morphine and then, once the dose has stabilized, I switch to therapy with an extended-release formulation
• Titration with an extended-release formulation, starting from the lowest dosage available for the chosen opiate
• I start with the dose that I think is appropriate for the clinical situation

27. How do I use opioids for the treatment of breakthrough pain (BTcP)?
• Title starting from low dosages
• I refer to dose proportional versus dose models of opioids used for background pain
• I start with the dose that I think is appropriate in relation to the type of pain and the clinical context

28. With which opiate do you think you can effectively initiate opioid therapy in a naïve patient?
• Opioid belonging to the second step of the WHO scale
• Immediate-release morphine
• Opioid belonging to the third step of the WHO scale
• I choose the opiate in relation to the type of pain and the clinical context

STRUCTURE

29. In the facility where you work, how is cancer pain managed?
• The patient is taken over by the specialist based on the pathology or treatment and referred to the specialist in pain therapy in difficult cases
• The patient is taken over by the specialist based on the disease or treatment and referred to the specialist in pain therapy only in advanced stages of the disease
• The patient is taken care of by the pain therapist from the onset of painful manifestations
Other (specify)

30. In the facility where you work, with which multi-professional pathway is cancer pain managed?
• The patient is managed by the pain therapist and several specialists without precise coordination
• The patient is managed according to a multidisciplinary approach (several specialists acting sequentially but coordinated)
• According to an interdisciplinary approach (several specialists acting simultaneously with periodic group re-evaluations)
• There is no multidisciplinary pathway
• Other (specify)

31. What about the timing when the pain therapist is involved with the oncologist? (Multiple answers are possible)
- When pain is assessed, regardless of the intensity or degree of the disease
- When the opioid dose exceeds 60 mg/day MME
- When there is severe pain > 7 (scale from 0 to 10)
- When the patient is destined for palliative therapy
- When pain is difficult to manage

32. Does the service also provide algological assistance at home?
Yes
No

33. Does the on-call anesthetist take care of urgent analgesic (oncological) consultations?
Yes
Yes, only at night and on holidays
No

34. Is there an availability for cancer patients at home? (More answers possible)
Yes
No
Daytime
Night
Holidays
Always

No availability

35. Are there beds in your structure dedicated to cancer pain therapy?
Yes, within the one-day care
Yes
No
Other (specify)

36. Are there priority criteria for access to the facility?
Yes, but the urgencies are delegated to the ED
Yes
Yes, but the system doesn't work
No

37. Is a periodic follow-up planned?
Yes
Yes, in accordance with precise clinical criteria
No

38. Does the follow-up of patients include the aid of telemedicine?
Yes
No
Not now, but it is being structured

39. In discharge, is the hospital supply of opioid drugs guaranteed for the continuation of therapy?
Yes
No

40. Is there an IT system that connects regional hospitals?
Yes
No

41. Is there an IT system that connects hospitals and home care?
Yes
No

42. Is there a digital computer system that connects General Practitioners and home care?
Yes
No

43. Is there a digital information system that connects General Practitioners and hospitals?
Yes

No

Structural equipment (from the pain centers census)

44. Indicates the technological resources present in the Pain Therapy Center: (Multiple answers are possible)
☐None
☐Echograph shared with other services
☐ Dedicated echograph
☐ Radiological section shared with other services
☐Dedicated radiological section
☐ Scrambler equipment
☐Magnetotherapy equipment
☐Radio frequency equipment
☐Laser therapy equipment
☐Other

45. What invasive-minimally invasive procedures are provided? (More answers are possible)
☐Intra-articular infiltrations
☐Laser therapy and / or other physical treatments
☐Spinal regional blocks
☐Spinal transforaminal blocks
☐Spinal catheterization
☐Perivenous blocks / catheterism
☐Peripheral, ganglion, trigeminal thermo-rhizotomies
☐Spinal neuromodulation
☐Peripheral spinal ganglion neurostimulation
☐Microdiscectomy
☐Diagnostic and therapeutic epiduroscopy
☐Radiofrequency of bone metastases
☐Neurolysis with pulsed frequency (facet joints)
☐Nucleoplasty
☐Discolysis
☐Kyphoplasty - Vertebroplasty
☐Position of systems for intrathecal injection of morphine / baclofen
☐Ganglionic lysis (alcohol)
☐None
☐Other

46. Were protocols adopted for the procedures selected in the previous question?
☐Yes, above all
☐Yes, on some

☐NO

47. Indicates the types of procedures for which the structures have declared that they have dedicated protocols: (multiple answers are possible)
☐ Outpatient
☐ Interventional / Surgical
☐ Diagnostics
☐ Other (specify)

48. Does the Pain Center adopt regional protocol?
☐YES
☐NO

49. Does the Pain Center adopt protocols for titration and use of Opioids?
☐YES
☐NO

50. Does the Pain Center adopt standardized protocols for opioid abuse cessation?
☐YES
☐NO

51. Does the Pain Center adopt standardized protocols for titration and use of Cannabinoids?
☐YES
☐NO

52. Does the Pain Center adopt an internal audit system aimed at verifying the adequacy of the therapeutic procedures used?
☐YES
☐NO
